# Investigating the prevalence of three medically important pathogens in *Ixodes pacificus* from southern Oregon

**DOI:** 10.3389/fpara.2025.1599377

**Published:** 2025-06-20

**Authors:** Andrew T. Partin, Emilio E. DeBess, Phillip Q. Spinks, Michael J. Yabsley, Kayla B. Garrett, James R. Clover, Geoffrey R. Taylor

**Affiliations:** ^1^ Jackson County Vector Control District, Central Point, OR, United States; ^2^ Public Health Division, Department of Human Services, Oregon Health Authority, Portland, OR, United States; ^3^ Placer Mosquito and Vector Control District, Roseville, CA, United States; ^4^ Southeastern Cooperative Wildlife Disease Study, College of Veterinary Medicine, University of Georgia, Athens, GA, United States; ^5^ Warnell School of Forestry and Natural Resources, University of Georgia, Athens, GA, United States; ^6^ Center for the Ecology of Infectious Diseases, University of Georgia, Athens, GA, United States; ^7^ Biological Consultant, Grants Pass, OR, United States

**Keywords:** ixodid, Pacific Northwest, tick-borne pathogens, western black-legged tick, zoonoses

## Abstract

**Introduction:**

In the far western United States of America, *Ixodes pacificus* is the primary vector of several pathogens of public health and veterinary importance including the Lyme disease spirochete *Borrelia burgdorferi* sensu lato (s.l.), as well as *Borrelia miyamotoi* and *Anaplasma phagocytophilum. Ixodes pacificus* is common in southern Oregon yet there are few published studies on the distribution of tick-borne pathogens in this region.

**Methods:**

Using real-time quantitative PCR, we assessed the prevalence of *B. burgdorferi* s.l., *B. miyamotoi*, and *A. phagocytophilum* among 2,463 unfed *I. pacificus* adults and nymphs combined into 260 pools (131 nymph, 129 adult) with nearly equal numbers of each life stage from 12 locations in Jackson County, Oregon.

**Results:**

In our study, 27.9% (36/129) and 29.8% (39/131) of adult and nymph pools, respectively, tested positive for at least a single pathogen. Nymph pools had a higher pool positivity rate (PPR) for *B. burgdorferi* s.l. with 15.3% (20/131) testing positive compared to 3.1% (4/129) of adult pools. Nymph pools also had a higher minimum infection rate (MIR) and maximum-likelihood estimate of pooled prevalence (EPP) for *B. burgdorferi* s.l. than adults. Interestingly, the prevalence of *B. burgdorferi* s.l. varied greatly in nymph pools across collection sites (0-70%). PPR of *B. miyamotoi* was 21.7% (28/129) for adults and 12.2% (16/131) for nymphs, making it the most frequently detected pathogen in adult pools and the most detected pathogen overall. *Anaplasma phagocytophilum* was the least frequently detected pathogen overall with a PPR of 3.1% (4/129) and 2.3% (3/131) for adults and nymphs, respectively.

**Discussion:**

These findings underscore the importance of continued surveillance, pathogen testing, and public education regarding ticks in areas such as southern Oregon where *I. pacificus* is common but little research has been done.

## 1 Introduction

The western black-legged tick, *Ixodes pacificus*, frequently bites humans and is of significant public health concern in the far western United States of America (USA), including the Pacific Coast states of Oregon, California, and Washington (Eisen et al., 2024). In this region, *I. pacificus* is the primary vector of multiple pathogens transmissible to humans and animals, including the Lyme disease spirochete *Borrelia burgdorferi* sensu lato (s.l.), *Borrelia miyamotoi*, and *Anaplasma phagocytophilum* (McVicar et al, 2022; Foster et al, 2023). Lyme disease, caused by the spirochete *B. burgdorferi* s.l., is the most common vector-borne disease in the USA (Diuk-Wasser et al., 2012). The incidence of Lyme disease is lower in Pacific Coast states with 0.2 cases per 100,000 persons compared to 30–80 cases per 100,000 persons per year in the Northeast and upper Midwestern states (Schwartz et al., 2017). Lower Lyme disease rates in Pacific Coast states are often attributed to the fact that in this region, lizards, primarily the western fence lizard (*Sceloporus occidentalis*) and southern alligator lizard (*Elgaria multicarinata*), serve as primary hosts for immature *I. pacificus* rather than mammals but are not competent reservoirs for *B. burgdorferi* s.l (Lane and Quistad, 1998; Wright et al., 1998; Lane et al., 2006). However, the results of studies examining the impact of lizards on the prevalence and distribution of Lyme disease are mixed.

For instance, a study that conducted an experimental removal of *S. occidentalis* found the density of infected nymphs (DIN) and, therefore, human risk of Lyme disease, was reduced after lizards were removed because ticks were not able to find an alternate, *Borrelia*-competent host in the absence of lizards (Swei et al., 2011). Other studies suggest lizards reduce Lyme transmission to humans by diverting feeding juvenile ticks away from reservoir-competent hosts such as the dusky-footed woodrat (*Neotoma fuscipes*), California kangaroo rat (*Dipodomys californicus*), and western gray squirrel (*Sciurus griseus*) (Lane and Brown, 1991; Salkeld et al., 2008). Therefore, tick hosts play an important role in pathogen transmission and prevalence since hosts differ in their ability to serve as competent reservoirs (Swei et al., 2011).

Despite the complex ecology of *B. burgdorferi*, it is generally accepted that nymphs are responsible for the majority of human Lyme disease infections (Barbour and Fish, 1993; Swei et al., 2011). In California, the majority of human Lyme disease cases occur during periods when nymphs are more active than adults (Ley et al., 1994; Clover and Lane, 1995; Salkeld et al., 2014). Additionally, several California studies of pathogen prevalence in *I. pacificus* have shown higher rates of *B. burgdorferi* s.l. in questing nymphs compared to adults (Burgdorfer et al., 1985; Bissett and Hill, 1987; Lane and Lavoie, 1988; Clover and Lane, 1995; Lane et al., 2010; Salkeld et al., 2014). A study in Washington found that 4.1% of host-seeking adult *I. pacificus* were infected with *B. burgdorferi* s.l. while 7.1% of nymphs were infected (Dykstra et al., 2020). The only available peer-reviewed study conducted in southern Oregon found that 3% of adult *I. pacificus* ticks pulled from rodents were infected with *B. burgdorferi* s.l. spirochetes (Burkot et al., 1999).

In 2013, *B. miyamotoi* was shown to cause disease in humans (Krause et al., 2013; Chowdri et al., 2013). The prevalence of *B. miyamotoi* and closely related relapsing-fever (RF) spirochetes in *I. pacificus* is generally between 1 and 2% but varies geographically (Barbour et al., 2009; Crowder et al., 2014). In California, infection rates of 1.6% and 1.7% were reported in *I. pacificus* nymphs from Mendocino and Sonoma counties, respectively, whereas adult infection rates were less <1% (Mun et al., 2006; Crowder et al., 2014). Another study in Mendicino County reported a 1.4% infection rate in *I. pacificus* nymphs (Lynn et al., 2018) which was similar to a statewide California study of *B. miyamotoi* prevalence (Padgett et al., 2014). In Washington, a study found *B. miyamotoi* in 4.4% of *I. pacificus* adults but no nymphs were positive (Dykstra et al., 2020). Unlike *B. burgdorferi*, which can only be transstadially transmitted, *B. miyamotoi* can also be transmitted vertically from female to larvae and was detected in all developmental stages of *I. pacificus* in a northern California study although prevalence was higher in adults and nymphs compared to larvae, suggesting that most transmission was horizontal (Sambado et al., 2020).

*Anaplasma phagocytophilum* has been considered a pathogen of veterinary importance since the 1930’s but was only recognized as a cause of human disease in 1994 (Chen et al., 1994). In North America, *I. scapularis* and *I. pacificus* are the most important vectors (Stuen et al., 2013) and several mammalian host species serve as competent reservoirs (Foley et al., 2008). Currently, there are few studies focusing on this pathogen in the far western USA. In a study conducted in Santa Cruz County, CA, a 6.19% *A. phagocytophilum* infection rate in adult *I. pacificus* was reported (Holden et al., 2003) while another study in the same county reported a 1% and 10% infection rate in nymphs and adults, respectively (Dingler et al., 2014). One study from Washington found a 1.9% *A. phagocytophilum* infection rate in *I. pacificus* adults while no nymphs in the study were positive (Dykstra et al., 2020).

Despite their importance as a vector of several human-infecting pathogens, published research regarding the distribution of *I. pacificus* and the prevalence of their associated pathogens in the state of Oregon is scarce. Our study assesses the prevalence of three important pathogens (*B. burgdorferi* s.l., *B. miyamotoi*, and *A. phagocytophilum*) in *I. pacificus* from 12 sites in Jackson County, Oregon to help address that gap. Our study provides baseline data regarding tick abundance and disease prevalence to allow for the monitoring of change over time and, importantly, we establish this data not only for adult *I. pacificus* but also for nymphs, which are regarded as epidemiologically more important.

## 2 Materials and methods

### 2.1 Field sampling

**Figure 1:**
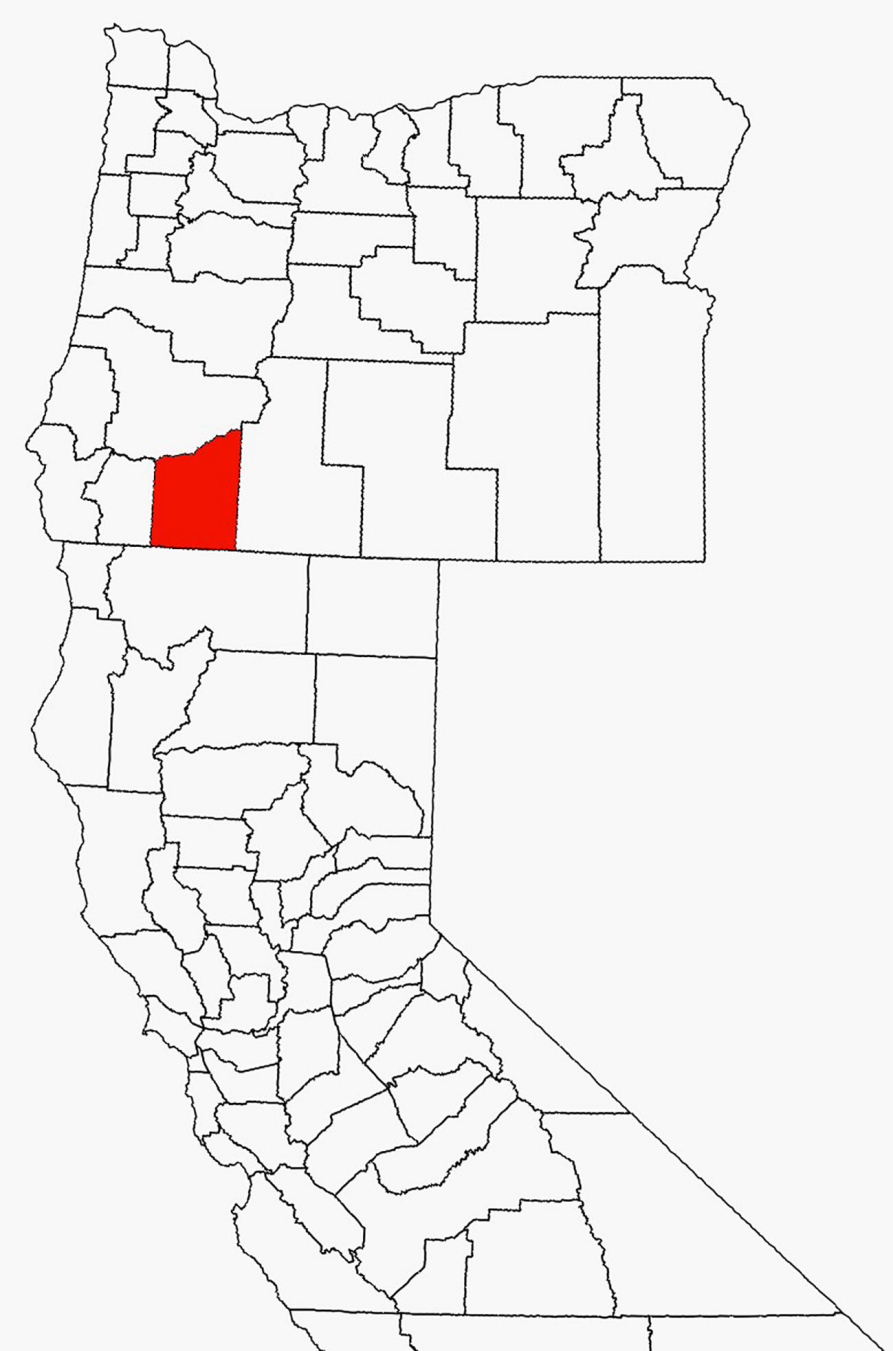
Map showing the location of Jackson County, Oregon, USA.

Tick collections were conducted in Jackson County, Oregon located along the northern border of California (**Figure 1**). Lyme disease risk has been shown to be relatively high in this region (Eisen et al., 2006). Adult ticks used in this study were collected by the Jackson County Vector Control District (JCVCD) from October to March 2022, 2023, and 2024 while nymphs were collected from April to June 2023. Our 12 study sites were chosen from among those used for prior adult tick collections. We excluded sites with dense understories and limited access to open areas; factors that would hinder nymph collection. We collected approximately 100 adults and 100 nymphs from each site which were all located on public land owned by federal, county, or city agencies. The habitat was either a mixed conifer or hardwood forest type. Questing adult *I. pacificus* were collected from the tips of grass and other vegetation along hiking trails, animal paths, and roadsides by sight and by flagging with 1-m² white double nap flannel cloth flags attached to 1.5-m wooden dowels. Nymphs were collected exclusively by flagging on and around downed trees, rotting logs, stumps, and other woody substrates since previous studies have shown that nymphs rarely climb vegetation while host seeking (Lane et al., 2007).

Because adult *I. pacificus* had been sampled at these sites by JCVCD prior to this study, each adult collection took less than two hours. In contrast, this was the first time nymphs had been sampled and the amount of time it took to collect enough specimens to meet the study goals was more varied, taking anywhere between two and four hours at each site. Nymph collections tended to take less time and effort at sites with easier access to downed woody debris. Also, in contrast to adult collections, some sites required two sampling events to collect an adequate number of nymphs.

As ticks were being collected in the field, they were stored together in 33mL polystyrene vials containing small pieces of grass for moisture. After each sampling event, those vials of ticks were placed inside resealable plastic bags labeled with collection site and date. A small piece of damp paper towel was placed inside each bag for additional moisture. Ticks were stored live in this manner for no more than one week in a standard refrigerator at 4°C until they could be processed further and separated into pools. Ticks were then counted and pooled by developmental stage and collection site. Adults were also pooled by sex. Pooled ticks were placed into 2ml Safe-Lock tubes (Eppendorf, Hamburg Germany) and stored dry at -80°C until they were sent for testing.

### 2.2 Statistical analyses

We used three methods to estimate pathogen prevalence in pooled specimens commonly found in the literature: pool positivity rate (PPR), minimum infection rate (MIR) and maximum-likelihood estimate of pooled prevalence (EPP) (Fracasso et al., 2023). PPR is defined and calculated as the ratio of positive pools to the total number of pools tested (Bertola et al., 2021). MIR is defined as the ratio of the number of positive pools to the total number of specimens tested while EPP is the infection rate most likely to be observed considering the tests results and an assumed probabilistic model (Cowling et al., 1999; Pilloux et al., 2015). To account for variable pool sizes, MIR and EPP estimates were calculated at a 95% confidence interval (CI) per 100 ticks using the software PooledInfRate v4.0 (Biggerstaff, 2009). Pearson’s Chi-Square tests were run using the Epitools epidemiological calculators https://epitools.ausvet.com.au/ (Sergeant, 2018) and were assessed at the 0.05 level to determine if differences in PPR were statistically significant for any pathogen between nymphs and adults as well as adult males and females.

### 2.3 Pathogen testing

For DNA extraction, ticks were pooled into 2ml Safe-Lock tubes (Eppendorf, Hamburg Germany) containing two 4 mm borosilicate glass beads along with 600 µl of grinding buffer composed of 3M guanidinium thiocyanate, 20 mM EDTA (pH 8), and 10 mM Tris-HCl (pH 8). Tick pools were homogenized for 60 seconds on an Omni Bead Ruptor Elite (Kennesaw, GA) then centrifuged at 14,000 G for 20 minutes. Next, 100 µl of supernatant of each pool was loaded in a deep well plate containing 600 µl extraction buffer (He et al., 2017) and 100 µl Sera-Mag™ SpeedBeads™ (Cytiva) that were prepared using a protocol maintained at OpenWetWare (https://openwetware.org/wiki/SPRI_bead_mix#Nucleic_acid_binding_bead_mixes). Extractions were carried out using the Opentrons OT-2 liquid-handling robot (Opentrons Inc., Brooklyn, NY) with an extraction protocol outlined in He et al.

A quantitative real-time PCR (qPCR) duplex assay for *B. burgdorferi* s.l. and *B. miyamotoi* has previously been described (Barbour et al., 2009) and here we expand the duplex assay into a triplex assay by including the primers and probe for *A. phagocytophilum* which were previously developed (Drazenovich et al., 2006). For our triplex assay, *A. phagocytophilum* was labeled with the Quasar 670™ dye while *B. burgdorferi* s.l. and *B. miyamotoi* were labeled with FAM and TAMRA reporter dyes, respectively. Primers and probes were obtained from BioSearch Inc. (Petaluma, CA). Quantitative PCR experiments were performed in 25 µl-volume reactions containing 10µl template, 4µl TaqMan™ Fast Virus 1-Step mastermix, and primer and probe concentrations at 900 nm and 200 nm, respectively. Cycling conditions were 50°C for 2 minutes and 95°C for 10 minutes, followed by 45 cycles of 95°C for 15 seconds and 64°C for 60 seconds on an Applied Biosystems QuantStudio™ 5 real-time detection system. Samples with cycle threshold (CT) values of < 40 were considered positive.

To determine the variant of *A. phagocytophilum* detected in ticks, an ~300 bp portion of the succinate dehydrogenase B560 subunit (SDHC) gene was amplified using primers TCGTCGGCAGCGTCAGATGTGTATAAGAGACAGAGTGTCTATAAGCTGCCGATAA and GTCTCGTGGGCTCGGAGATGTGTATAAGAGACAGAACATCAACCAACCACTGAA as described (Hojgaard et al., 2024). Amplicons were detected in a 1% agarose gel stained with GelRed (Biotium, Fremont, CA, USA) and extracted from the gel using a QIAGEN gel extraction kit (Germantown, MD, USA). Amplicons were bi-directionally sequenced at Genewiz (South Plainfield, NJ, USA). Sequences were edited and assembled, primer sequences removed, aligned with related sequences from GenBank, and a phylogenetic tree was constructed using an approximately maximum-likelihood method with FastTree v2.1 with a generalized time-reversible (GTR) model in Geneious Prime 2024.0.7 (Biomatters Limited, Auckland, New Zealand). Unique sequences were submitted to GenBank (PV768874-PV768876).

## 3 Results

Overall, 2,463 unfed *I. pacificus* ticks consisting of 1,246 adults (684 females, 562 males) and 1,217 nymphs were collected from 12 locations in Jackson County, Oregon and grouped into 260 pools of 131 nymph and 129 adult pools (**Table 1**). Most pools (70%) contained 10 specimens, but pool size ranged from 4–16 individuals across the study. The number of pools from each site for each life stage varied from 10-15. In our study, 27.9% (36/129) and 29.8% (39/131) of adult and nymph pools, respectively, tested positive for at least a single pathogen. *Borrelia burgdorferi* s.l. was the most frequently detected pathogen in nymphs but PPRs for this pathogen were low at most collection sites (~0-10%); however, nymph pools from Roxy Ann Peak, Sterling Mine Ditch trail, and Forest Park had higher PPRs for this pathogen (70%, 60%, and 30%, respectively) (**Table 2**). For *B. burgdorferi* s.l., nymphs had a PPR of 15.3% (20/131) while adult pools had a PPR of 3.1% (4/129) (**Table 3**). For *B. burgdorferi* s.l., nymph pools had an EPP of 1.77 (1.12-2.68 CI) and a MIR of 1.64 (0.93-2.36 CI) compared to adult pools which had an EPP of 0.32 (0.11-0.78 CI) and a MIR of 0.32 (0.01-0.64 CI) (**Table 4**).

**Table 1:** Overview of sites where *I. pacificus* were collected in 2022–2024 from Jackson County, Oregon.

Site	Coordinates	Elevation (m)	No. of adults	No. of nymphs
(1) Crowfoot Rd	42.61853 -122.68819	602	117	100
(2) Felton Trail	42.30111 -123.17358	748	100	100
(3) Forest Park	42.31800 -122.99900	587	100	100
(4) Lithia Park (Hitt Rd)	42.19056 -122.72632	816	104	100
(5) Lower Table Rock	42.46687 -122.94665	392	104	100
(6) Roxy Ann Peak	42.35505 -122.79203	898	100	100
(7) Sterling Creek	42.24500 -122.97800	921	100	100
(8) Sterling Mine Ditch Trl.	42.18660 -122.95012	784	100	100
(9) Upper River Road	42.44732 -123.01050	375	105	100
(10) W. Fork Evans Creek	42.59500 -123.01800	458	106	100
(11) Woodrat Mountain	42.23100 -123.00400	1,159	100	117
(12) Yellow Rock Trl.	42.69175 -122.72704	521	110	100
Total			1246	1217

**Table 2:** Number of positive pools and pool positivity rate (PPR)% by collection site.

Site	Adults					Nymphs				
	No. pools	No. ticks	*B. burgdorferi*	*B. miyamotoi*	*A. phagocytophilum*	No. pools	No. ticks	*B. burgdorferi*	*B. miyamotoi*	*A. phagocytophilum*
1	11	117	1 (9.1)	1 (9.1)	1 (9.1)	10	100	0	0	0
2	10	100	0	2 (20)	10	15	100	0	3 (20)	0
3	10	100	0	3 (30)	0	10	100	3 (30)	4 (40)	2 (20)
4	10	104	0	2 (20)	0	11	100	1 (9.1)	0	0
5	11	104	1 (9.1)	2 (18.2)	1 (9.1)	10	100	1 (10)	1 (10)	0
6	13	100	1 (7.7)	3 (23.1)	0	10	100	7 (70)	1 (10)	0
7	10	100	0	4 (40)	0	10	100	0	2 (20)	0
8	10	100	0	2 (20)	0	10	100	6 (60)	3 (30)	0
9	12	105	0	2 (16.7)	0	10	100	1 (10)	0	0
10	10	106	0	1 (10)	1 (10)	10	100	1 (10)	0	0
11	10	100	1 (10)	2 (20)	0	12	117	0	0	0
12	12	110	0	4 (33.3)	0	13	100	0	2 (15.4)	1 (7.7)
Total	129	1246				131	1217			

**Table 3:** Number of positive pools and pool positivity rates (PPR)% of *Borrelia burgdorferi, Borrelia miyamotoi*, and *Anaplasma phagocytophilum* DNA within *I. pacificus* adult and nymph pools.

Stage	No. ticks	No. pools	No. positive pools and PPR (%)		
			*Bb*	*Bm*	*Ap*
Nymphs	1217	131	20 (15.3)	16 (12.2)	3 (2.3)
Adults	1246	129	4 (3.1)	28 (21.7)	4 (3.1)

**Table 4:** Maximum-likelihood estimate of pooled prevalence (EPP), minimum infection rate (MIR) and confidence intervals (CI) for *I. pacificus* adult and nymph pools.

Stage	Pathogen	EPP with lower and upper limit (95% CI)	MIR with lower and upper limit (95%CI)
Nymphs	*B. burgdorferi*	1.77 (1.12-2.68)	1.64 (0.93-2.36)
	*B. miyamotoi*	1.38 (0.82-2.18)	1.31 (0.67-1.95)
	*A. phagocytophilum*	0.25 (0.07-0.67)	0.25 (0-0.53)
Adults	*B. burgdorferi*	0.32 (0.11-0.78)	0.32 (0.01-0.64)
	*B. miyamotoi*	2.53 (1.72-3.60)	2.25 (1.42-3.07)
	*A. phagocytophilum*	0.32 (0.11-0.78)	0.32 (0.01-0.64)

The PPR of *B. miyamotoi* was 21.7% (28/129) for adults and 12.2% (16/131) for nymphs, making it the most frequently detected pathogen in adult pools and the most detected pathogen overall (**Table 3**). At least one adult pool from each collection site was positive for *B. miyamotoi* (**Table 2**). The EPP of *B. miyamotoi* was 2.53 (1.72-3.60 CI) for adult pools compared to 1.38 (0.82-2.18 CI) for nymphs. MIR for *B. miyamotoi* was also higher for adult pools compared to nymphs (**Table 4**). *A. phagocytophilum* was the least frequently detected pathogen in this study with PPRs of 3.1% (4/129) and 2.3% (3/131) for adults and nymphs, respectively (**Table 3**). *A. phagocytophilum* had the lowest EPP and MIR estimates for all target pathogens but these estimates were slightly higher for adults (**Table 4**).

**Figure 2:**
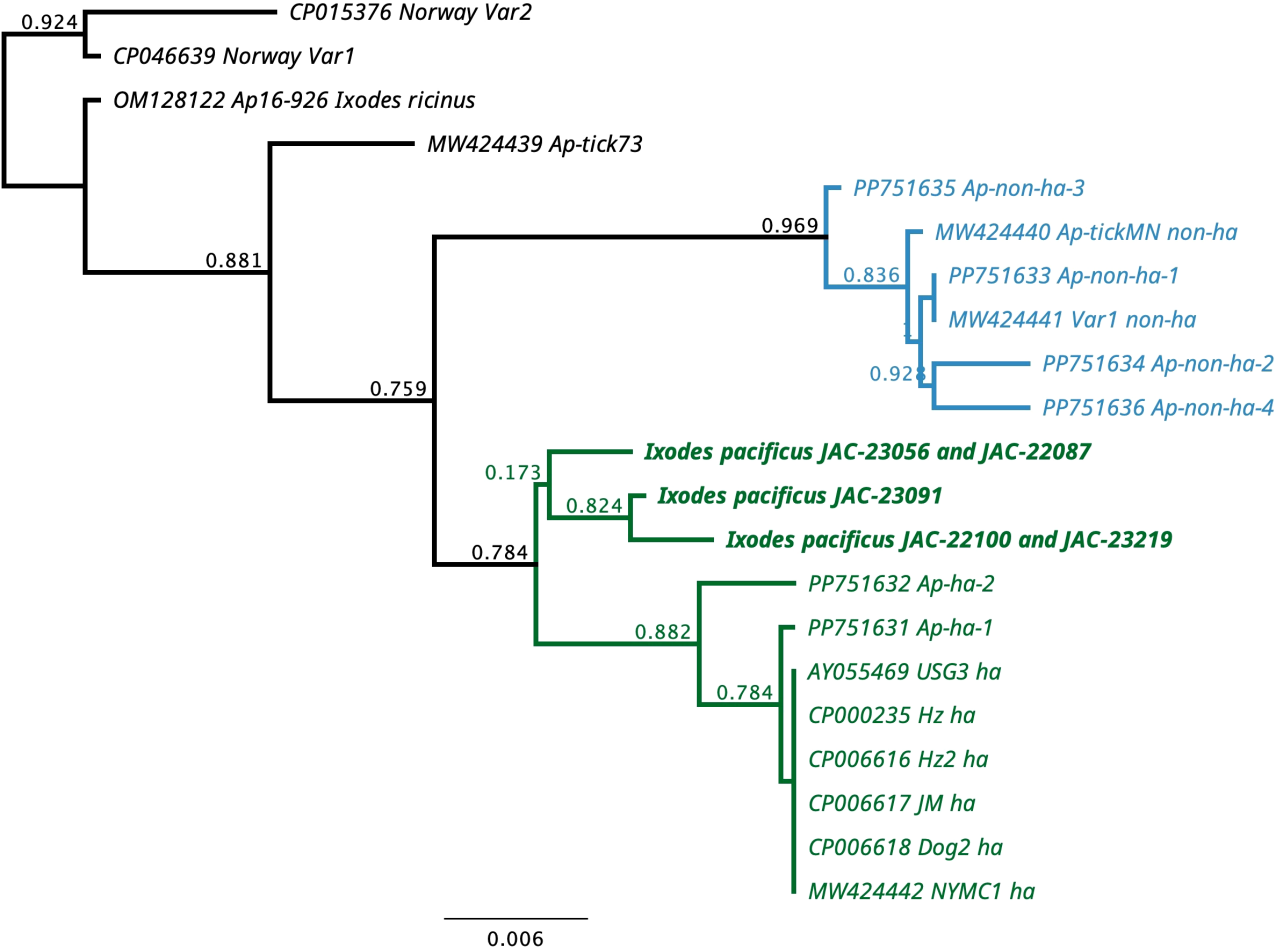
Phylogenetic tree for 296 bp of the succinate dehydrogenase B560 subunit (SDHC) gene of *Anaplasma phagocytophilum* from *Ixodes pacificus* from Oregon, USA and representative sequences of human-infective (ha) and non-human-infective (non-ha) strains of *A. phagocytophilum*.

A total of five ticks initially detected as positive for *A. phagocytophilum* were positive with the SDHC gene PCR. Three unique sequences were obtained, and they were 99%-99.7% similar to each other and 98-98.7% similar to numerous ha-strains of *A. phagocytophilum* from humans, domestic dogs, and *I. scapularis* (**Supplementary Table 1**). The Oregon *A. phagocytophilum* sequences were only 96.1-97.3% similar to non-ha strains from white-tailed deer (*Odocoileus virginianus*) and *I. scapularis* (**Supplementary Table 1**). Phylogenetically, the Oregon sequences grouped together and were in a clade with several human-infecting strains (**Figure 2**).

A Pearson’s Chi-Square test conducted at the 0.05 level determined the difference in PPR between nymphs and adults for *B. burgdorferi* s.l. was statistically significant (*df* = 4, *P* = 0.009). Differences in PPR between developmental stages were not statistically significant for *B. miyamotoi* (*df =* 4, *P =* 0.228) or *A. phagocytophilum* (*df =* 4, *P =* 0.955). Differences in PPR between adult males and females were not statistically significant for *B. burgdorferi* s.l. (*df* = 4, *P* = 0.651), *B. miyamotoi* (*df =* 4, *P =* 0.512), or *A. phagocytophilum* (*df =* 4, *P =* 0.757). Additionally, our study found that 3.5% (9/260) of pools were positive for multiple pathogens. Four adult pools and three nymph pools were coinfected with *B. burgdorferi* s.l. and *B. miyamotoi* while one adult pool and one nymph pool were coinfected with *B. miyamotoi* and *A. phagocytophilum*.

## 4 Discussion

Although *I. pacificus* is commonly found in southern Oregon, little is known about the distribution and prevalence of tick-borne pathogens in the region. We assumed all *Ixodes* ticks collected for our study were *I. pacificus*, although two previous studies documented low totals of *Ixodes* sp*inipalpis* and *Ixodes angustus* infesting small mammals in southern Oregon (Burkot et al., 1999; Xu et al., 2019). However, these species live almost exclusively inside the nests or burrows of their host and are not known to quest on exposed vegetation in warm, low humidity climates. Instead, they prefer cooler and wetter conditions found along the Pacific coast (Lane and Keirans, 1999; Gregson, 1956). *Dermacentor* spp. were also collected as bycatch while flagging but were discarded. Testing of nearly 2,500 unfed *I. pacificus* adults and nymphs for select pathogens found that *B. burgdorferi* s.l., *B. miyamotoi*, and *A. phagocytophilum* were all present. Sequence analysis of the SDHC confirmed that five of the *A. phagocytophilum* samples were the human-variant.

An important difference between this study and most studies previously conducted in the far western USA, is that our study tested ticks in pools rather than individually. Pool screening is helpful in reducing processing times, limiting the cost of diagnostic testing, and has been an accepted way to analyze arthropod vectors in human and veterinary medicine for decades (Dorfman, 1943; Farrington, 1992). However, compared to testing specimens individually, pool screening has limitations, and the accuracy of results depend greatly on the pooling strategy used. Pooling strategy is important for a number of reasons. First, the true number of infected specimens cannot be determined in pools that test positive for a pathogen (Furstenau et al., 2020). Secondly, high numbers of specimens in a pool, low infection prevalence, or high engorgement status can affect accuracy. Third, pools consisting of specimens from different areas or that were collected at different times can affect results. Lastly, if pools contain mixed life stages, results can be biased and make comparison difficult since different life stages have different chances of being infected (Fracasso et al., 2023). To minimize these issues, we limited the number of specimens per pool, and separately pooled specimens by life stage and sex.

In addition to the way specimens are pooled, the statistical methods used to estimate prevalence can also introduce bias and impact accuracy. We used three methods to estimate prevalence found in the literature: pool positivity rate (PPR), minimum infection rate (MIR), and maximum-likelihood estimate of pooled prevalence (EPP). PPR is the most commonly used index but it does not estimate the number of infected individuals in a pool; it is simply the ratio of positive pools to the total number of pools tested (Fracasso et al., 2023). MIR is a widely used method for estimating infection prevalence in pooled samples but it is also influenced by pool size and is only capable of estimating the lower limits of an infection rate because it assumes only a single infected specimen exists in a positive pool (Cowling et al., 1999; Hojaard et al., 2024). For estimating true individual infection prevalence, EPP is considered superior to MIR because it better accounts for confounding effects and covariates through the use of statistical software packages (Biggerstaff, 2009; McLure et al., 2021; Fracasso et al., 2023). To help account for variable pool sizes in the study, we used a PooledInfRate software in Excel to estimate both MIR and EPP at a 95% CI.

PPR was higher than MIR and EPP estimates in adults and nymphs for all pathogens while estimates of MIR and EPP were similar across life stages and pathogens (**Tables 3**, **4**). This is consistent with another study that compared methods for estimating pathogen prevalence in pooled specimens (Fracasso et al., 2023). Although it is difficult to draw direct inferences between this study and those that test ticks individually, our results showed, for *B. burgdorferi* s.l., all three of these calculations were higher for nymphs compared to adults (**Tables 3**, **4**). For nymph pools, the EPP for *B. burgdorferi* s.l. was 1.77 (1.12-2.68 CI) compared to 0.32 (0.11-0.78 for adult pools. The difference in PPR for *B. burgdorferi* s.l. between nymphs (15.3%) and adults (3.1%) was statistically significant but was not for other pathogens. These numbers suggest that in southern Oregon, like California, questing *I. pacificus* nymphs are typically infected with *B. burgdorferi* s.l. at higher rates than adults and are therefore medically more important (Burgdorfer et al., 1985; Lane and Lavoie, 1988; Clover and Lane, 1995; Burkot et al., 1999; Lane et al., 2007; Salkeld et al., 2021). Interestingly, nymph pools at three sites, all heavily trafficked public use trails, had considerably higher PPRs (30-70%) compared to other sites where PPR was lower (0 -10%) (**Table 2**). Previous studies have found wide variations in tick-borne pathogen prevalence between collection sites, emphasizing the importance of reporting infection prevalence not only on a state or regional scale, but also at site level (Salkeld et al., 2021; Foster et al., 2023). Such variation in PPR is important from a human risk standpoint because it suggests the probability of contracting Lyme disease may vary greatly on a local scale in Jackson County and perhaps in other nearby regions. Future studies investigating host ecology in these areas to determine which species are being utilized by immature ticks would be beneficial to determine why higher PPRs were observed.

*Borrelia miyamotoi* was the most frequently detected pathogen in adult pools and was the most common pathogen overall in adult and nymph pools combined (**Table 3**). It was found in at least one adult pool from every collection site (**Table 2**). This evidence shows this pathogen is readily common in both nymphs and adults and is widely distributed in Jackson County. MIR and EPP estimates for *B. miyamotoi* in this study (**Table 4**) were similar to individual infection rates reported in several previous studies from California and Washington (Mun et al., 2006; Xu et al., 2019; Dykstra et al., 2020). This evidence also suggests that there may be significantly more human cases of *B. miyamotoi* in Jackson County than are currently known.

Our study did confirm the presence of *A. phagocytophilum* in Jackson County in both nymphs and adults. *Anaplasma phagocytophilum* was the least commonly detected pathogen in this study and all estimation indexes were closer between adults and nymphs for this pathogen than for any other (**Tables 3**, **4**). Prevalences between 0.3 – 2.0% have been reported from Oregon and California (Xu et al., 2019; Eshoo et al., 2015; Pascoe et al., 2019). Only 1 and 2 cases of anaplasmosis per million people in Oregon and California respectively were reported via the National Electronic Telecommunications System for Surveillance (NETSS) and the Centers for Disease Control and Prevention (CDC) case report form (CRF) (Demma et al., 2005). Although human cases occur more frequently in the coastal northeast and northern Midwest regions of the US and cases have increased nationwide from 2012-2016 (Baker et al., 2020), the low prevalence of *A. phagocytophilum* in our study seemingly correlates with low incidences of anaplasmosis reported in the far western USA. The SDHC sequences we detected were unique but clustered with the human-infecting variants of *A. phagocytophilum*. The genetic variation we detected could be due to lack of sequences from the western United States (Hojgaard et al., 2024).

Our study also found that 3.5% (9/260) of pools were coinfected with multiple pathogens. Although this presents the possibility of coinfections in individual ticks, making this determination is beyond the scope of our study. Additional work needs to be done in our region testing individual ticks for pathogens as opposed to pooled specimens. This could have epidemiological importance since humans can become infected with multiple pathogens if bitten by a coinfected *Ixodes* tick, potentially increasing the severity of disease and making diagnosis, as well as treatment, more difficult (Belongia, 2002).

Our results confirm the presence of three medically important pathogens in *I. pacificus* adults and nymphs from southern Oregon. This highlights the need for continued disease testing and surveillance of *I. pacificus* in southern Oregon, as well as other parts of the state where this species is common but little research has been done to determine the distribution or prevalence of their associated pathogens. This type of proactive monitoring is crucial for protecting public health by allowing us to better understand and anticipate the risk of tick-borne diseases, particularly in understudied regions where this risk may actually be relatively high. Our findings also suggest it would be helpful to conduct future studies regarding host ecology at field sites where higher PPRs were observed, particularly *B. burgdorferi* s.l., to determine if host species at those specific sites could be causing higher infection rates in ticks. In addition to increased disease testing and surveillance of ticks and tick hosts, our results should emphasize the importance of raising public awareness of ticks and tick-borne diseases whether or not local public health agencies have any active surveillance programs in place.

## Data Availability

The original contributions presented in the study are publicly available. This data can be found here: NCBI GenBank, accession PV768874-PV768876.
